# Optimization of Nutritional and Sensory Properties of Unleavened Flatbread Developed From Maize‐Boiled Rhizome of Water Lily—Fish

**DOI:** 10.1002/fsn3.70192

**Published:** 2025-04-22

**Authors:** Alemu Lema Abelti, Tilahun A. Teka, Geremew Bultosa

**Affiliations:** ^1^ Department of Postharvest Management, College of Agriculture and Veterinary Medicine Jimma University Jimma Ethiopia; ^2^ Batu Fish and Other Aquatic Life Research Center Oromia Agricultural Research Institute Batu Ethiopia; ^3^ Department of Food Science and Technology Botswana University of Agriculture and Natural Resources Gaborone Botswana

**Keywords:** boiled rhizome of water lily, dried fish fillet powder, maize, unleavened flatbread

## Abstract

The aim of this study was to optimize nutritional composition, mineral contents, and overall sensory acceptability of unleavened flatbread developed from maize, boiled rhizome of water lily, and dried fish fillet powder. A mixture containing 50%–80% maize, 15%–30% boiled water lily rhizome, and 5%–20% dried fish fillet powder was mixed using the D‐optimal mixture design. Flatbread developed from maize flour was used as the control. The results showed that the linear model described well changes in crude protein, total carbohydrate, gross energy value, sodium, potassium, iron, and zinc. The lack of fit was non‐significant (*p* > 0.05) for all the linear models, indicating that the linear model equations fit the data well. The addition of boiled rhizome of water lily flour and dried fish fillet powder to maize improved protein content, potassium, calcium, magnesium, iron, and zinc but decreased fat, fiber, carbohydrate, and gross energy value. Numerical optimization results showed that a blending ratio containing 62.62% maize, 29.92% boiled rhizome of water lily, and 7.45% dried fish fillet powder resulted in the best formulation with a desirability function value of 0.516. It can be concluded that this bread can potentially reduce protein energy malnutrition and micronutrient deficiencies.

## Graphical abstract

Our study revealed that the blending ratio containing 62.62% of maize flour, 29.92% of boiled rhizome of water lily flour, and 7.45% of fish flour had the best formulation with a desirability function value of 0.516, with enhanced protein, gross energy value, potassium, iron, zinc, and overall sensory acceptability of flatbreads.

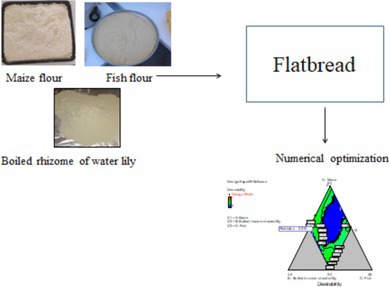

## 1 Introduction

Protein energy malnutrition, stunting, and micronutrient deficiencies are the most significant risk factors for diseases and mortality in children, particularly in least developed countries. Fortification of staple food using a variety of ingredients is the best way to address nutrient deficiencies (Cardoso et al. 2019). Staple food becomes nutritionally dense when fortified with protein‐rich underutilized ingredients. In Ethiopia, especially in the Rift Valley, traditional unleavened flatbread constitutes staple bakery foods (Diddana et al. 2021).

Maize is the major ingredient used to prepare traditional unleavened flatbread in Ethiopia. Maize was selected as one ingredient to develop flatbread because it is an important crop which adapt to central rift valley and higher yield potential (Markos et al. 2024). Additionally, maize is used as a raw material to make several staple foods, including traditional unfermented bread (locally known as ‘kita’ or ‘bixillee’), traditional porridge (‘ganfo’ or ‘marqaa’), and maize flour (‘duket’ or ‘daakuu’) (Nyachoti et al. 2021). Unleavened flatbread is circular bread baked on a hot iron griddle. Because it is simple to prepare, flatbread is eaten for meals three times a day in many areas. Unleavened flatbread is distinct from other ready to eat snacks in the central Rift Valley of Ethiopia due to its larger consumer base and greater convenience to prepare (Diddana et al. 2021).

Maize contains protein (7%–13%), fat (1.4%–6%), and carbohydrate (74%–80%). However, because maize could not provide enough micro and macronutrients to meet human nutritional needs, it is a food of a low nutritional value. Fortification of maize with fish has been the basis of efforts to increase the amount of insufficient amino acids in maize, hence improving its nutritional value (Goredema‐Matongera et al. 2021).

Fish contains essential amino acids that are limiting in cereals, roots, and tubers. Fish is known to contain polyunsaturated fatty acids (EPA‐Eicosapentaenoic acid and DHA‐Docosahexaenoic acid) and two essential amino acids (methionine and lysine) (Mendivil 2021). Hence, food containing fish, tubers, and maize is complementary toward fulfilling human dietary requirements. The fish species selected as an ingredient was common carp ( *Cyprinus carpio* ). Abelti and Teka (2024) recently reported that common carp ( *C. carpio* ) is underutilized and is not in high demand. Consequently, this fish is vulnerable to massive (21%) postharvest losses (Abelti and Teka 2024).

There is a need to make use of this underutilized fish species as an ingredient for the development of protein‐rich and easily digestible traditional unleavened flatbread. It is important to enrich flatbread using common carp, which has a low market price as compared to warm‐water fish due to its unpleasant smell and dichotomous bony nature of its flesh (Khodaveisi et al. 2024). Hence, the addition of common carp flesh to cereals and tubers is an important strategy to increase the usage of fish fillet in the human diet. The presence of essential amino acids in fish that are limited in maize makes fish an ideal ingredient to improve the nutritional value of maize. The fortification of maize with fish flour would improve the overall essential amino acid balance and aid in the fight against protein energy malnutrition. Additionally, fortification can enhance the nutritional quality and shelf life of fish products like powder and chapatti (Mekonnen and Aychiluhm 2024).

The staple foods in the tropics are crops like cereals, roots, and tubers. They provide 75% of the total energy and 67% of total protein intake. However, these crops are low in nutritional value as they are insufficient in macro and micro nutrients (FAO 2017). There is also a wild edible aquatic plant that can be used as a source of protein and energy (Chawanje et al. 2001). Notably, Afrin et al. (2024) reported that the rhizome of the water lily can be used as an ingredient to make value‐added bakery products. A bakery product like a biscuit has been developed by adding 30% of the rhizome of the water lily to wheat (Afrin et al. 2024). Incorporating tubers of *N. lotus* with wheat flour increased the protein, fat, and fiber contents, making it a viable ingredient for the preparation of biscuits (Adanse et al. 2021). It was reported that the rhizome and seed of the water lily can complement conventional cereals consumed by human beings (Danhassan et al. 2018; Gueye et al. 2020).

The flour of water lily rhizome contains protein (8.57%), carbohydrate (67.7%), and fiber (9.78%); as a result, it could be used as a dietary supplement (Afrin et al. 2024). Out of the ten essential amino acids that need to be supplied through diets, six essential amino acids are found in the rhizome of *N. lotus* . Particularly, two of the rare amino acids (lysine and methionine) are present in relatively high amounts; as a result, the rhizome of *N. lotus* is nutritionally valuable (Hujjatullah et al. 1967). We recently reported that the rhizome of *N. lotus* contains protein (23.6%), carbohydrate (61.7%), lysine (3.4 g/100 g of protein), and methionine (1.4 g/100 g of protein) (Abelti et al. 2024). Additionally, the rhizome of water lily is a rich source of starch (Abelti, Teka, and Bultosa 2024a, 2024c).

In Ethiopia, the rhizome of *N. lotus* is locally known as ‘kinta’, ‘moche’, or ‘kurumbo’. This rhizome is collected and consumed by cattle herding children and not used for sale (non‐commercial). The nutrient, anti‐nutrient, phytochemical, amino acids, and elemental contents of this rhizome makes it a good dietary source that can be utilized to make functional food (Abelti et al. 2024b, 2023).

Traditional unleavened flatbread has been chosen for this study due to its widespread acceptability, increased consumers, availability of maize, ease of preparation, affordability, and low cost. Despite there being detailed studies on phytochemicals, the acute toxicity test of its crude extract, proximate compositions, and antioxidant properties of *N. lotus* , no data is available regarding the use of rhizome, maize, and fish for making flatbread. Hence, the water absorption capacity, oil absorption capacity, bulk density, tapped density, compressibility index, Hauner ratio, and sensory properties of both composite flour and flatbread of rhizome, fish, and maize have not been studied so far. The objective of this study was to optimize flatbread rich in protein and energy from maize, boiled rhizome of water lily, and fish and determine the proximate compositions, mineral contents, and overall sensory acceptability of flatbread.

## 2 Materials and Methods

### 2.1 Sampling and Sample Preparation

Three ingredients (maize, water lily rhizome, and fish) were used to prepare unleavened flatbread. The whole fish of Common carp ( *C. carpio* ) was purchased from the local market. The fish specimen was filleted using a stainless steel knife. Immediately after filleting, it was cool‐transported using an ice box to the Batu Fish and other aquatic life research center laboratory. The fillet was dried for 2 h at 90°C and finally for 6 h at 60°C (Komolafe et al. 2013). The dried fillet was finally ground with a laboratory miller (high speed multi‐functional crusher Al Marwani for spice, Model:400A, Shanghai, China) into fine powder and stored in a polythene bag for further analysis.

**FIGURE 1:**
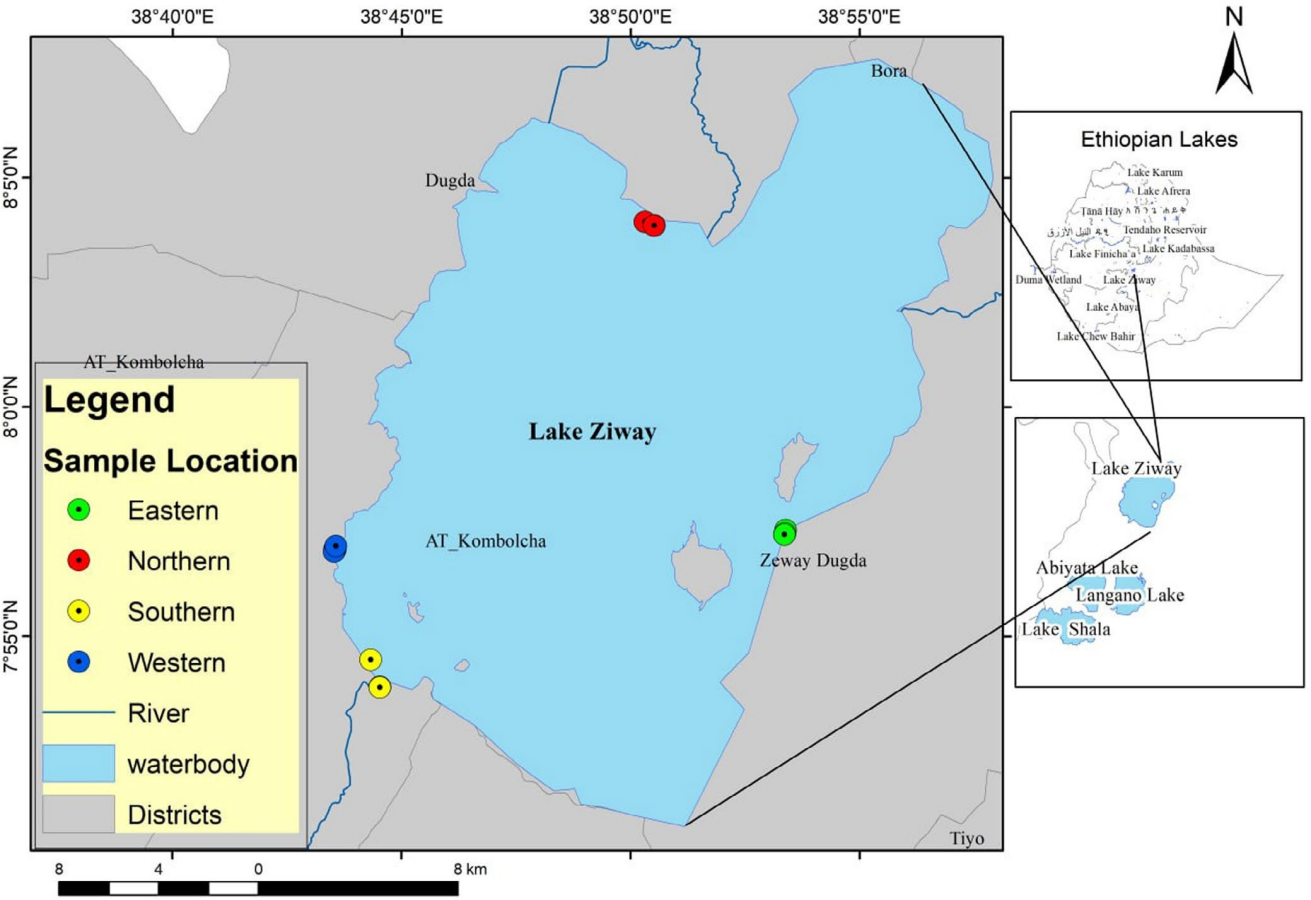
Maps of Lake Ziway showing the study area.

A 20 kg of 124‐b (113) maize variety was collected from the Bako Agricultural Research Center. The maize was cleaned of extraneous materials and milled using a laboratory miller (high speed multi‐functional crusher Al Marwani for spice, Model:400A, Shanghai, China) and sieved using 0.2 mm size. The rhizome of *N. lotus* was collected from four areas of Lake Ziway (Figure 1) according to the procedure described in Abelti, Teka and Bultosa (2024a). The rhizome was cooked and finally dried for 3 days at 60°C (Danhassan et al. 2018). The dried rhizome was ground to a fine powder using a laboratory miller (high speed multi‐functional crusher Al Marwani for spice, Model:400A, Shanghai, China), sieved using a 2 mm mesh sieve, and then stored in zip‐lock plastic bags for further analysis.

### 2.2 Experimental Design

A total of eleven experimental runs were generated using D‐optimal constrained mixture design with Design expert 7.0.0 version. The design of the experiment was based on three components consisting of maize flour (MF), boiled rhizome of water lily flour (BRWLF), and dried fish fillet powder (FF) with the sum of the components equal to 100%. A constrained mixture design was employed as it is not possible to produce flatbread with 100% rhizome or fish. The component ranges were as follows: 0.5 ≤ MF ≤ 0.8, 0.15 ≤ BRWLF ≤ 0.3, 0.05 ≤ FF ≤ 0.2 (Table 1). These constrained ranges were set based on previous studies on value‐added products prepared (bread and cookies) from wheat and boiled rhizome of water lily. Maize flour was used as a control.

**TABLE 1:** Percentage of three ingredients used to prepare traditional unleavened flatbread.

Sample code	Maize flour	Boiled rhizome of water lily flour	Fish flour
MRF‐1	79.99	15.00	5.00
MRF‐2	56.95	28.89	14.15
MRF‐3	65.50	15.00	19.49
MRF‐4	64.84	30.00	5.15
MRF‐5	72.14	20.50	7.35
MRF‐6	58.43	21.56	20.00
MRF‐7	79.99	15.00	5.00
MRF‐8	64.10	23.46	12.4
MRF‐9	65.50	15.00	19.49
MRF‐10	69.37	25.62	5.00
MRF‐11	50.03	30.00	19.96
Maize	100	—	—

The linear and quadratic models were used to fit the response values to the generated data. The criteria used to choose the predictive model for each response were analysis of variance (ANOVA) at a *p*‐value of *p* ≤ 0.05 and a *p*‐value of lack of fit at 0.05. Numerical optimization was carried out to select the best blending ratio of maize, boiled rhizome of water lily, and dried fish fillet powder in accordance with the recommendations of World Health Organization 2004, National Health and Medical Research Council of Australia and New Zealand, 2006, and the European Union's Regulation (EC) No 1924/2006 on foods. Water absorption capacity, oil absorption capacity, bulk density, tapped density, protein, fiber, carbohydrate, energy, potassium, iron, zinc, and overall sensory acceptability of flatbread were maximized. In contrast, Carr's compressibility index, Hausner ratio, moisture content, fiber, ash, and sodium were minimized.

### 2.3 Preparation of Traditional Unleavened Flatbread

Traditional unleavened flatbread was prepared using the straight dough method according to Saeed et al. (2021). The ingredients required for developing traditional unleavened flatbread maize—flour, boiled rhizome of water lily flour, dried fish fillet powder, and distilled water (110 mL) were mixed and made into dough in the mixing bowl. All the ingredients were mixed for 15 min in a bowl to obtain dough. The control flatbread was prepared from maize flour alone. The dough was rolled into a circular disk‐like shape to appropriate diameter and thickness, and the dough was baked at 180°C using a baking oven (Baking oven Kumtel, Model: KF‐5320, Ethiopia) until an attractive color and aroma were released. The baked traditional unleavened flatbread was cooled at room temperature, dried, milled using a laboratory miller (high speed multi‐functional crusher Al Marwani for spice, Model:400A, Shanghai, China), and packed using polyethylene bags for analysis.

### 2.4 Functional Properties of Composite Flour

#### 2.4.1 Water Absorption Capacity

Water absorption capacity (WAC) of the composite flour was determined according to the procedure of Kaushal et al. (2012). Briefly, flour (1 g) was measured into a pre‐weighed centrifuge tube; water (10 mL) was added and centrifuged (Table top centrifuge, Model: PLC‐Gemmy industrial corp., Taiwan) at 4000 rpm for 10 min. The supernatant was decanted and measured using a 10 mL graduated cylinder. The WAC of the flour was calculated as follows:

$$\mathrm{WAC}\left(\frac{g}{g}\right)=\frac{\left(\text{Weight of centrifuge tubes after decant}-\text{weight empty centrifuge tubes}\right)-\text{weight of sample}\;}{\text{Weight of the sample}}$$

#### 2.4.2 Oil Absorption Capacity

Oil absorption capacity (OAC) of the composite flour was determined according to the procedure of Kaushal et al. (2012). Accordingly, flour (1 g) was measured into a pre‐weighed centrifuge tube, vegetable oil (10 mL) was added, and centrifuged (Table top centrifuge, Model: PLC‐Gemmy industrial corp., Taiwan) at 4000 rpm for 10 min. The unbound oil was decanted and measured using a 10 mL graduated cylinder. The OAC of the flour was calculated as follows:

$$\mathrm{OAC}\left(\frac{g}{g}\right)=\frac{\left(\text{Weight of centrifuge tubes after decant}-\text{weight of}\;\text{empty centrifuge tubes}\right)-\text{weight of sample}\;}{\text{Weight of the sample}}$$

#### 2.4.3 Bulk Density and Tapped Density

Bulk density (BD) of the composite flours was measured according to the procedure of Kaushal et al. (2012). Briefly, a flour sample (10 g) was slowly filled into a graduated cylinder (25 mL) and the volume occupied by the flour was recorded. In the case of tapped density, the bottom of the graduated cylinder was tapped 500 times on the laboratory bench until there was no change in the volume occupied by the filled flour. The bulk density and tapped density were calculated as follows:

$$\text{Bulk density}\;\left(\frac{g}{\mathrm{mL}}\right)=\frac{\text{Sample weight}}{\text{Volume occupied}\;\mathrm{by}\;\text{the filled flour}\;\left(\mathrm{mL}\right)}$$

$$\begin{array}{c} \text{Tapped density}\;\left(\frac{g}{\mathrm{mL}}\right)\\ =\frac{\text{Sample weight}}{\text{Volume occupied}\;\mathrm{by}\;\text{the filled flour after tapping}\;\left(\mathrm{mL}\right)} \end{array}$$

#### 2.4.4 Compressibility Index and Hausner Ratio

Both compressibility index and Hausner ratio were calculated taking into account both bulk density and tapped density according to the method of Manek et al. (2012):

$$\text{Compressibility index}\;\left(\%\right)=\frac{\text{Tapped density}-\text{Bulk density}}{\text{Tapped density}}\times 100$$

$$\text{Hausner ratio}=\frac{\text{Tapped density}}{\text{Bulk density}}$$

#### 2.4.5 Proximate Compositions of Flatbread

The moisture content, crude protein content, crude fat, crude ash, and crude fiber were determined according to AOAC (2015) official method numbers. The carbohydrate content was calculated by subtracting the sum of moisture, protein, fat, ash, and fiber from 100.

$$\begin{array}{c} \text{Carbohydrate}\;\left(\%\right)=100\\ -\left[\text{moisture}\;\left(\%\right)+\text{protein}\;\left(\%\right)+\mathrm{fat}\;\left(\%\right)+\mathrm{ash}\;\left(\%\right)+\text{fiber}\;\left(\%\right)\right] \end{array}$$

The gross energy value (kcal/100 g) was calculated according to Atwater conversion factors.

$$\begin{array}{c} \text{Gross energy value}\;\left(\text{kcal}/100\;g\right)\\ =4*\text{crude protein}+4*\text{carbohydrate}+9*\mathrm{fat} \end{array}$$

#### 2.4.6 Mineral Contents of Flatbread

The mineral contents (K and Na) and (Ca, Mg, Zn, and Fe) were determined using a flame photometer (Flame photometer, Model: FP 910, pg. instruments, United Kingdom) and flame atomic absorption spectroscopy (Varian Spectra AA‐20 Plus, Varian Australia Pty. Ltd., Australia), respectively according to Diddana et al. (2021). Stock solutions of sodium and potassium and nitrate's salt of calcium, magnesium, zinc, and iron were used as standard solutions. A series of working standard solutions were prepared and used to construct standard curves using absorbance versus concentration. The mineral contents (mg/100 g) were determined according to the following equation:

$$\text{Minerals content}\;\left(\frac{\mathrm{mg}}{100\;g}\right)=\frac{\left(A-B\right)\times V}{10*\text{Sample weight}}$$

where *A* = concentration of sample solution, *B* = concentration of blank, *V* = volume of the extract.

#### 2.4.7 Sensory Properties of Flatbread

The organoleptic properties of traditional unleavened flatbreads were evaluated using a nine‐point hedonic scale. Accordingly, 50 untrained consumers who were familiar with the consumption and quality aspects of unleavened flatbreads were randomly selected from the Batu Fish and other Aquatic Life Research center staff. The flatbread was presented on a white plate and assigned a four‐digit code of which the evaluators were unaware of the proportion and types of ingredients used (blind test) and served to the consumers along with a glass of water. The water was provided so that the untrained panelists could rinse their mouths in between each evaluation, which took 10 s. The consumers evaluated (1 extremely dislike and 9 extremely like) texture, color, taste, aroma, and overall acceptability. The mean sensory scores for each attribute were plotted using a radar chart.

#### 2.4.8 Data Analysis

The data of functional properties of flour, proximate composition, and minerals content were presented using grand mean. The overall sensory acceptability of the flatbread was presented using a radar chart. Analysis of variance (ANOVA) was used to examine the significance of linear and quadratic models for each response variable. Design expert version 7.0.0 was used to construct the contour plots. Finally, numerical optimization was carried out to determine the optimum blending ratio of maize, boiled rhizome of water lily, and dried fish fillet powder based on the recommendation to minimize or maximize the response variables. Pearson's correlation was used to determine the association between the analyzed parameters.

## 3 Results and Discussion

### 3.1 Model Selection

The analysis of variance (ANOVA) *p*‐values for functional properties of composite flours, proximate composition, mineral contents, and overall sensory acceptability are presented in Table 2. The results showed that the linear model described well the changes in crude protein, total carbohydrate, gross energy value, sodium, potassium, iron, and zinc (*p* < 0.05). Both linear and quadratic models did not well describe water absorption capacity, oil absorption capacity, tapped density, Hausner ratio, moisture content, crude fat (CF), total fiber (TF), and overall sensory acceptability (*p* > 0.05). Hence, the grand mean well described the changes of WAC, OAC, TD, HR, MC, CF, TF, and OSA. The quadratic models have predictive power to express the changes in Carr's compressibility index and bulk density (*p* < 0.05).

**TABLE 2:** Analysis of variance (ANOVA) for functional properties, proximate composition, mineral contents, and overall sensory acceptability.

Source	WAC	OAC	BD	TD	CI	HR	MC	CP	CF	TA	TF	TC	GEV	Na	K	Fe	Zn	OSA
Linear	0.591	0.277	0.05	0.469	0.644	0.792	0.614	0.031	0.501	0.729	0.232	0.05	0.05	0.03	0.009	0.032	0.009	0.576
A × B	0.430	0.321	0.06	0.442	0.116	0.720	0.770	0.544	0.713	0.734	0.713	0.548	0.550	0.551	0.596	0.545	0.418	0.179
A × C	0.815	0.978	< 0.0001	0.462	0.113	0.074	0.332	0.318	0.516	0.885	0.516	0.416	0.229	0.320	0.299	0.322	0.481	0.086
B × C	0.565	0.901	< 0.0001	0.974	0.02	0.125	0.278	0.832	0.435	0.774	0.435	0.884	0.254	0.824	0.731	0.834	0.854	0.709
Adj. *R* ^2^	0.350	0.604	0.9833	0.352	0.718	0.545	0.432	0.768	0.499	0.162	0.499	0.726	0.748	0.768	0.855	0.764	0.852	0.577
Lack of fit	0.133	0.476	—	0.425	0.371	0.335	0.967	0.740	0.634	0.683	0.634	0.989	0.641	0.744	0.745	0.749	0.687	0.762

*Note:* A, total ash; Adj. *R* ^2^, adjusted coefficient of determination; BD, bulk density; CF, crude fat; CI, compressibility index; CP, crude protein; Fe, iron; GEV, gross energy value; HR, Hausner ratio; K, potassium; MC, moisture content; OAC, oil absorption capacity; OSA, overall sensory acceptability; TC, total carbohydrate; TD, tapped density; TF, total fiber; WAC, water absorption capacity; Zn, zinc.

The non‐significant (*p* > 0.05) lack of fit for all the linear models indicates the linear model well fit to the data of bulk density, crude protein, total carbohydrate, gross energy value, sodium, potassium, iron, and zinc (*p* < 0.05) for the prepared traditional unleavened flatbread. The regression equation of the selected models for each response variable was examined to predict the parameter presented in Table 3. From 18 parameters of both flour and flatbread analysis carried out, only the linear regression of eight response variables was significant. Hence, the following linear regression equations were used to predict the corresponding response values.

**TABLE 3:** Regression equation of the selected models for each response.

Response variables	The selected regression equation model
Bulk density	*Y* = 0.506*X*_1_ + 1.05*X*_2_ + 4.92*X*_3_
Crude protein	*Y* = 12.23*X*_1_ − 136.33*X*_2_ + 436.17*X*_3_
Total carbohydrate	*Y* = 62.79*X*_1_ + 208.61*X*_2_ − 220.3*X*_3_
Gross energy value	*Y* = 3330.35*X*_1_ + 89.26*X*_2_ − 306.18*X*_3_
Sodium	*Y* = −273*X*_1_ − 501.1*X*_2_ + 886.6 *X*_3_
Potassium	*Y* = 265.26*X*_1_ − 440.68*X*_3_ + 3418.42*X*_3_
Iron	*Y* = 1.22*X*_1_ − 19.8*X*_2_ + 61.16*X*_3_
Zinc	*Y* = 1.86*X*_1_ + 3.58*X*_2_ + 0.408*X*_3_

Abbreviations: *X*_1_, maize flour; *X*_2_, boiled rhizome of water lily flour; *X*_3_, fish flour; *Y*, response variables.

### 3.2 Functional Properties of Composite Flours

The functional properties of composite flours are intrinsic characteristics that can influence how food systems behave while being processed and stored. Sufficient understanding of these attributes demonstrates flour's usefulness and acceptability.

#### 3.2.1 Water Absorption Capacity (WAC)

**FIGURE 2:**
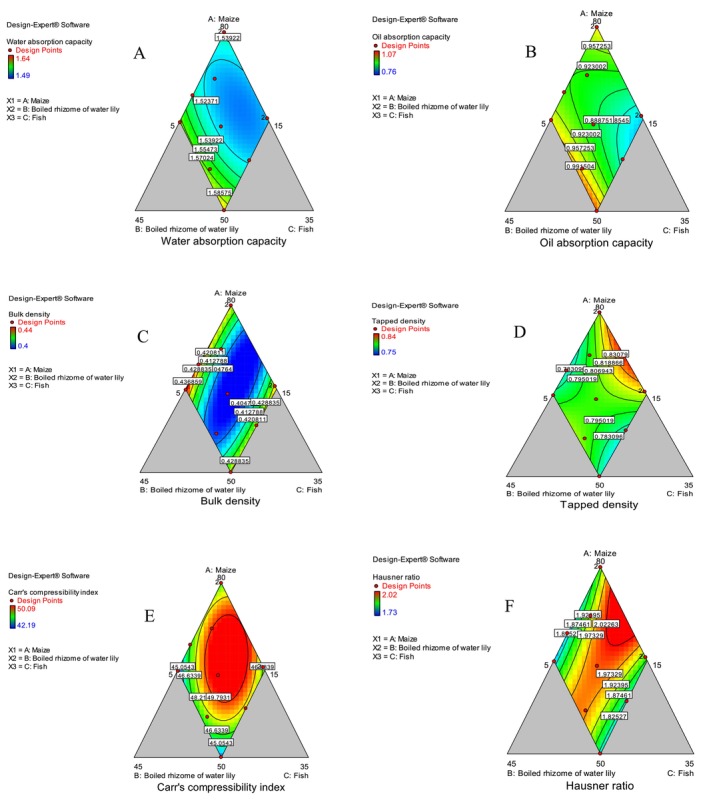
Contour plot showing functional properties (A—water absorption capacity, B—oil absorption capacity, C—bulk density, D—tapped density, E—Carr's compressibility index, and F—Hausner ratio).

The WAC of composite flours is presented in Table 4. The composite flours were significantly (*p* < 0.05) different in terms of WAC. Even though the ANOVA results showed that both the linear and quadratic models were non‐significant (*p* > 0.05), the increase in water absorption capacity is attributed to an increase in the boiled rhizome of water lily and a decrease in maize flour (Figure 2A). The lowest WAC (1.49 g/g) was obtained from MRF‐6 flour, whereas the highest WAC (1.64 g/g) was obtained in MRF‐11.

**TABLE 4:** Functional properties of composite flours.

Sample code	WAC (g/g)	OAC (g/g)	BD (g/mL)	TD (g/mL)	CI (%)	HR
MRF‐1	1.54	1.04	0.43	0.78	45.17	1.82
MRF‐2	1.50	0.96	0.40	0.81	50.09	2.02
MRF‐3	1.55	0.92	0.43	0.83	47.25	1.89
MRF‐4	1.60	0.92	0.44	0.76	42.99	1.73
MRF‐5	1.57	0.91	0.41	0.82	50.09	2.00
MRF‐6	1.49	0.76	0.43	0.75	48.82	1.75
MRF‐7	1.53	0.95	0.43	0.84	47.96	1.95
MRF‐8	1.55	0.92	0.40	0.78	48.60	1.92
MRF‐9	1.50	0.79	0.43	0.83	45.58	1.94
MRF‐10	1.50	1.02	0.43	0.81	47.08	1.89
MRF‐11	1.64	1.07	0.43	0.79	42.19	1.83
Maize	1.30	0.93	0.43	0.76	42.85	1.75

*Note:* BD, bulk density; CI, compressibility index; HR, Hausner ratio; OAC, oil absorption capacity; TD, tapped density; WAC, water absorption capacity.

The increase in the WAC of composite flour is attributed to the increase in protein content (Hasmadi et al. 2020). The WAC of flour can be affected by the amounts of carbohydrate and protein present in the flour. The flour of the boiled rhizome of water lily and dried fish fillet powder has a higher protein content as compared to maize flour alone. MRF‐11 contained a blending ratio of higher boiled rhizome of water lily flour (30%) and dried fish fillet powder (20%). The difference in WAC of the composite flours is due to the variations in nature and types of proteins found in the three ingredients (maize, boiled rhizome of water lily, and fish) (Tiencheu et al. 2016).

Fiber, in addition to protein, is reported to contribute to the water holding and retention capacities of flours (Godswill 2019). Flour having a low WAC like MRF‐6 is useful when making thinner gruels or porridges since more flour can be added per unit volume of gruel, boosting the infant foods' energy and nutrient density (Godswill 2019). Contrary to this, a high WAC like MRF‐11 is useful for the preparation of bakery products that need to be hydrated for their handling and textural qualities benefit (Godswill 2019).

#### 3.2.2 Oil Absorption Capacity (OAC)

Oil absorption capacity is defined as the physical entrapment of oils due to the binding of protein to fat during food formulation. The OAC of composite flours is presented in Table 4. The lowest OAC (0.76 g/g) was recorded from MRF‐6, and the highest OAC (1.07 g/g) was obtained from MRF‐11. The increased maize flour ratio decreased oil absorption capacity (Figure 2B). Fish flour has been reported to contain greater OAC (2.4 g/g) as compared to maize flour (1.6 g/g) (Lema 2018). The higher oil absorption capacity in MRF‐11 indicates the presence of high hydrophobic proteins in composite flour as compared to other blending ratios. The oil retaining properties of food materials can be affected by both the content and types of proteins (Ravi and Sushelamma 2005). Oluwalana et al. (2011) reported that flour can absorb more oil when non‐polar amino acids are exposed to it. Capillary attraction is the mechanism by which fat is physically bound to proteins which contribute to oil absorption capacity.

Previous study reported that the blending ratio containing high fermented tilapia had a higher oil absorption capacity as compared to the blending ratio containing lower fermented tilapia and maize (Fasasi et al. 2007). Generally, the oil absorption capacity of the composite flours was lower than the water absorption capacity of their corresponding composite flours due to the fact that more hydrophilic interactions were present in the composite flour (Tiencheu et al. 2016). Oil absorption capacity plays an important role in developing new products, enhancing flavor and mouth feel. The relatively high OAC of the MRF‐11 blend indicates that it could be useful in food formulation where oil absorption capacity is required, such as in bakery products (Godswill 2019).

#### 3.2.3 Bulk and Tapped Densities of Composite Flours

The bulk and tapped densities, compressibility index, and Hausner ratio of composite flours are presented (Table 4). The bulk density of a powder is the ratio of the mass of an untapped powder sample to its volume. The highest bulk density (0.44 g/mL) was obtained from MRF‐4, and the lowest was obtained from MRF‐2 and MRF‐8. The ANOVA results indicated that the linear model significantly (*p* < 0.05) described changes in bulk density, but the quadratic model (maize and boiled rhizome of water lily) did not significantly (*p* > 0.05) describe changes in bulk density. As a result, the bulk density of composite flour increased with an increase in boiled rhizome of water lily (Figure 2C). The present finding was in agreement with a previous study that reported that the bulk density of maize (0.48 g/g) and fish (0.43 g/g) were comparable (Lema 2018). Particle size and moisture content affect the bulk density of composite flours (Hasmadi et al. 2020). The bulk density result indicated that samples MRF‐2 and MRF‐8 had more moisture than the other composite flours. As compared to the present finding, Fasasi et al. (2007) reported a slightly lower bulk density of flour composted from maize and tilapia, which ranged from 0.355 g/mL to 0.384 g/mL.

Flours with high bulk density are appropriate for reducing paste thickness. Flour having a high bulk density is required for ease of dispensability and to reduce paste thickness (Sengev et al. 2012). The value of bulk density of flour is important to determine the types of packaging materials required for storing and transporting the composite flours. Contrary to this, flours having a low bulk density (MRF‐2 and MRF‐8) are advantageous for formulating complementary foods because it increases nutrient and calorie per feed of child (Dzandu et al. 2025; Abeshu et al. 2016). Flour containing fish as an ingredient was reported to reduce bulk density and is desirable to reduce viscosity, which is important for the formulation of high density weaning foods and gruels (Fagbemi et al. 2005).

The tapped density is obtained by mechanically tapping a graduated measuring cylinder or vessel containing the powder sample. In Table 4, the tapped bulk density is presented. The lowest tapped density (0.75 g/mL) and the highest tapped density (0.84 g/mL) of composite flour were obtained in MRF‐6 and MRF‐7, respectively. The increases in maize and fish flours have increased the tapped density of composite flours (Figure 2D). The tapped density of fish (0.66 g/g) was reported to be higher than the tapped density of maize (0.52 g/g) (Lema 2018). It was observed that the values for tapped density for various samples were more than their respective values for bulk density. The present finding is in line with the results of Tiencheu et al. (2016), who reported 0.8–0.83 g/mL of tapped density for the composite flours of maize, pawpaw, red beans, and mackerel fishmeal. Contrary to this, Fasasi et al. (2007) reported lower tapped density (0.513 to 0.61 g/mL) for maize and tilapia composite flours. Determining a powder's tapped density is critical because it tells us how well the particles can pack together under mechanical compaction, which gives us information about the powder's flowability, compressibility, and suitability for processing. In other words, it tells us how much volume a given mass of powder will occupy after being tapped tightly.

#### 3.2.4 Carr's Compressibility Index and Hausner Ratio

The Carr compressibility index and Hausner ratio are measures of the product's ability to settle and also provide information about flowability and cohesiveness behavior of the flour samples. The lowest Carr's compressibility index (42.19%) was calculated from MRF‐11, and the highest compressibility index (50.09%) was calculated from MRF‐2 and MRF‐5 (Table 4). Carr index measures the relative significance of inter particle interaction. In the constrained region of experimental area, the CI has increased with neither increment nor decrement ratio of all three ingredients (Figure 2E). Carr's compressibility index is used in food and pharmaceuticals as an indicator of the flowability of flour.

The highest Hausner ratio (2.02) was calculated from MRF‐2, while the lowest Hausner ratio (1.73) was calculated from MRF‐4. The Hausner ratio determines the flowability nature of a powder or granules. The Hausner ratio of composite flour has increased with an increase in the maize flour ratio (Figure 2F). The values of the Hausner ratio and Carr's index were used to classify the flour sample compression, compaction, and flowability as excellent, good, fair, or poor (Carr, 1965). The first‐ever study on CI and HR was carried out by Carr; even though it is too old, CI and HR are important information for describing the flow properties of flours.

Composite flours having Carr's index < 10, 11–15, 16–20, 21–25, 26–31, 32–37, and > 38 are classified as flowability of excellent, good, fair, passable, poor, very poor, and non‐flowing (Carr 1965). The composite flours having Hausner ratio 1.00–1.11, 1.12–1.18, 1.19–1.25, 1.26–1.34, 1.35–1.45, 1.46–1.59, and > 1.60 are classified as flowability of excellent, good, fair, passable, poor, very poor, and non‐flowing (Carr 1965). Accordingly, all the composite flours prepared from maize, boiled rhizome of water lily, and fish can be classified as non‐flowing flours according to Carr's (1965) classifications. The higher values of CI and HR indicated the cohesive nature of the flours, which may lead to incorrect flows, bridging, and discharge problems in hoppers (Kaushik et al. 2024).

### 3.3 Proximate Compositions of Flatbread

**FIGURE 3:**
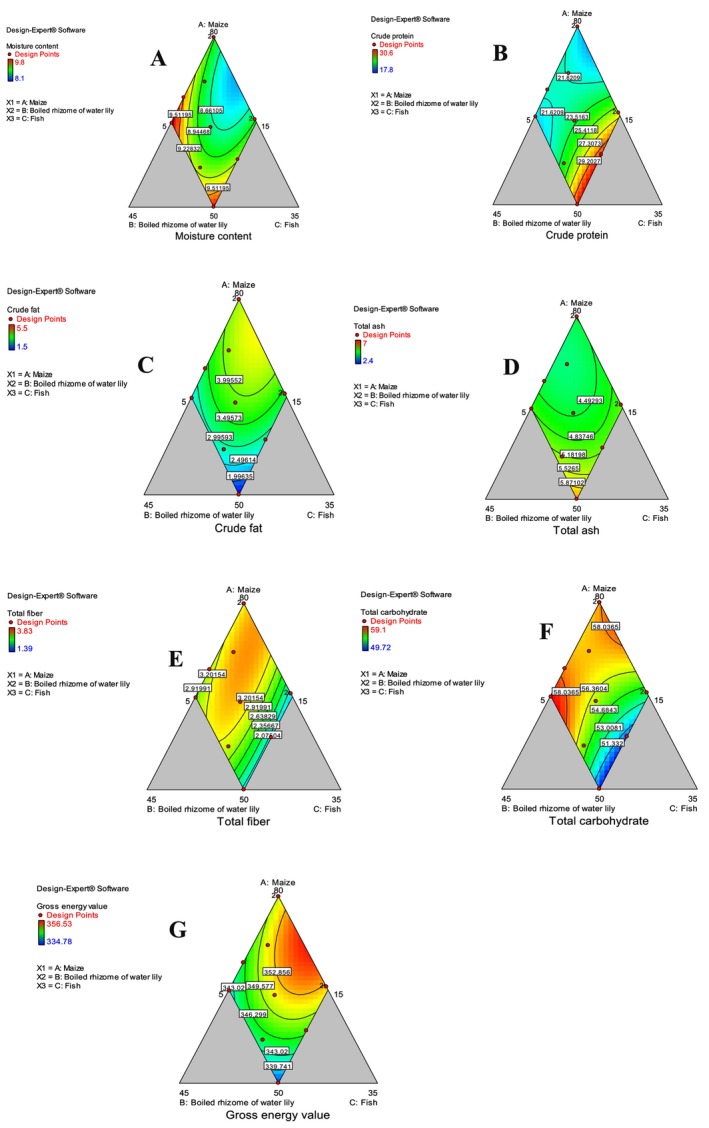
Contour plot showing proximate compositions (A—moisture content, B—protein content, C—fat content, D—ash, E—fiber, F‐carbohydrate, and G‐gross energy value).

In Table 5, the moisture content, crude protein, crude fat, total ash, total fiber, total carbohydrate, and gross energy value of traditional unleavened flatbread are presented. The lowest moisture content (8.1%) was obtained in MRF‐9, whereas the highest moisture content (9.8%) was equally obtained in MRF‐3, MRF‐10, and MRF‐11 of the flatbreads (Table 5). There was a decrement in moisture content of flatbread as the ratio of maize and fish flours decreased in the composite flours (Figure 3A).

**TABLE 5:** Proximate composition of traditional unleavened flatbread.

Sample code	Moisture content (%)	Crude protein (%)	Crude fat (%)	Total ash (%)	Total fiber (%)	Total carbohydrate (%)	Gross energy value (kcal/100 g)
MRF‐1	9.4	17.8	4.5	4.5	3.2	59.1	348.1
MRF‐2	9.2	25.0	3.0	5.0	2.3	55.4	348.8
MRF‐3	9.8	24.3	2.2	3.1	2.5	58.0	348.4
MRF‐4	9.7	19.4	3.0	6.0	3.2	58.7	339.4
MRF‐5	8.6	18.6	5.5	5.5	3.8	56.5	349.8
MRF‐6	9.1	29.8	3.0	6.0	1.6	50.4	347.9
MRF‐7	8.6	23.4	3.5	4.5	3.1	56.8	352.5
MRF‐8	8.8	24.2	3.0	4.0	3.8	56.2	348.5
MRF‐9	8.1	28.9	4.5	7.0	1.4	50.1	356.5
MRF‐10	9.8	24.3	2.6	3.0	2.7	58.5	348.5
MRF‐11	9.8	30.6	1.5	6.0	2.4	49.7	334.8
Maize	8.8	12.4	8.5	3.0	4.8	62.5	376.1

The moisture content of the present study is higher than the one reported by Saeed et al. (2021). These authors reported the moisture content of 3.36% for 5% lotus root flour substituted with wheat flour and a moisture content of 4.53% for 25% lotus root flour substituted with wheat flour. Similar to this study, Afrin et al. (2024) reported 2.38%, 3.4%, and 2.34% moisture content in biscuits developed from 0%, 20%, and 30% *N. lotus* tuber flours substituted with wheat flour, respectively. The moisture content of all the flatbread samples had moisture contents that were within acceptable ranges for long‐term storage and in line with acceptable standards. According to Codex Alimentarius Commission, the maximum permissible moisture is set at 15.5% (Bodor et al. 2024). Flours having moisture content higher than 15.5% are more prone to contamination, which can lead to mycotoxin infections and unpleasant odors (Bodor et al. 2024).

The protein content of the flatbread varied between different blending ratios (Table 5). The protein content of the flatbread developed from the composite flour ranged from 17.8% (MRF‐1) to 30.6% (MRF‐10). It was observed from Table 2 that only the linear model significantly (*p* < 0.05) described well the changes in protein due to blending, while the quadratic as well as the cubic models were non‐significant, implying that the model did not describe the changes in protein. The proportion of maize flour, boiled rhizome of water lily flour, and fish flour has a positive linear effect on crude protein content at (*p* < 0.05). The protein content of the flatbread was increased with the increment of fish and boiled rhizome of water lily and the decrement of maize flour (Figure 3B). A previous study indicated that the protein content (75.9% db) of fish flour was reported to be higher than the protein content of maize (12.4%) (Lema 2018). From the present study, it was observed that the protein content was increased in all the unleavened flatbreads as compared to flatbread prepared from maize alone.

The study regarding the addition of water lily rhizome flour to maize flour to developed bakery product is limited. However, information regarding the incorporation of water lily rhizome flour to wheat flour for developing biscuits or bread is available (Afrin et al. 2024; Ibrahim 2007). The addition of 20% and 30% water lily tuber flour to wheat flour increased the protein content of biscuits to 7.22% and 9.52%, respectively (Afrin et al. 2024). In a similar study, Adanse et al. (2021) observed that the highest protein content was recorded in flour containing 60% wheat flour, 25% *N. lotus* flour, and 15% coconut flour. This urges the need for fortification of maize flour with fish flour (Fasasi et al. 2007). According to National Health and Medical Research Council of New Zealand and Australia recommendation, children and adolescents aged 2 to 17 should consume 14 to 45 g of protein each day. The protein contents of all the prepared flatbread fulfilled this requirement and within the permissible ranges.

World Health Organization (2004) recommends that infants and young children should feed on a formulation with a protein level of food that can provide at least 15% total energy, which is met by all of the prepared unleavened flatbread in this study. In another regulation, food is claimed to be a source protein if at least 20% of the energy value of food is provided by protein (Regulation (EC) No 1924/2006). Similarly, a study in agreement with the above findings indicated that incorporation of 10%, 15%, and 20% of *N. lotus* rhizome flour into wheat improved the protein content of bread to 13.5%, 13.4%, and 13.3%, respectively (Ibrahim 2007). Adanse et al. (2021) also observed that the addition of 6%, 12%, 18%, and 25% *N. lotus* tuber to wheat improved the protein content of flour to 12.01%, 15.76%, 16.11%, and 16.73%, respectively.

The presence of fats and/or oils in foods lowers the overall volume of the food ingested while increasing the energy density and quantity of fatty acids. It is preferable to have at least 20% of energy come from fat. The ANOVA result has shown that both linear and quadratic models were not significant (*p* > 0.05) to describe the changes of fat due to the blending ratio (Table 2). The crude fat content of unleavened flatbread is presented in Table 5. The highest and lowest crude fat were obtained as 5.5% and 1.5% in MRF‐5 and MRF‐11, respectively. The additions of boiled rhizome of water lily and fish flours have reduced the fat content of unleavened flatbread (Figure 3C) as fat was not obtained in the rhizome of water lily (Abelti et al. 2024b). Flatbread with low fat content is desirable as high fat affects the shelf life of the flatbread due to oxidative rancidity (Awolu et al. 2016). Food is claimed to be low fat if the food contains no more than 3 g/100 g ((EC) No 1924/2006). All the prepared flatbreads except MRF‐1, MRF‐5, MRF‐7, MRF‐9, and MRF‐11 are considered to be low fat foods.

The ash content of the traditional unleavened flatbread is presented in Table 5. The lowest ash content (3%) was observed in MRF‐10 and the highest ash content (7%) was observed in MRF‐9. The results of ANOVA indicated that both the linear and quadratic models had not significantly (*p* < 0.05) described the changes in total ash content (Table 2). The addition of boiled rhizome of water lily flour and fish flour, as well as the decrement of maize ratio, has increased the ash content of flatbread developed from the composite flour (Figure 3D). The total ash content of fish flour (6.4%) is higher than the total ash content of maize flour (1.2%) (Lema 2018).

The present study is in agreement with Afrin et al. (2024), who observed that the addition of water lily tuber flour to wheat increased the ash content of biscuits. The study indicated that the ash content of biscuits developed from 0%, 20%, and 30% flour of *N. lotus* tuber substituted with wheat was 2.38%, 1.89%, and 2.26%, respectively (Afrin et al. 2024). The increment of crude ash in blended flatbread as compared to maize bread gave a clear indication of high minerals in fish flour and boiled rhizome of water lily.

The total fiber content ranged from 1.4% (MRF‐9) to 3.8% (MRF‐5 and MRF‐8) as presented in Table 5. The ANOVA results showed that both the linear and quadratic models were not significant (*p* > 0.05), they did not describe the changes in fiber due to varying blending ratios (Table 2). The total fiber in the flatbread is attributed to the fiber content of maize flour and boiled rhizome of water lily flour. Naturally, fish has no fiber. Food is claimed to be high in fiber if it contains at least 3 g of fiber per 100 g (Regulation (EC) No 1924/2006). Some of the flatbreads (MRF‐1, MRF‐2, MRF‐5, MRF‐7, and MRF‐8) prepared can meet the dietary fiber requirements of human beings.

The total carbohydrate content of the flatbread ranged from 49.7% (MRF‐11) to 59.1% (MRF‐1) as indicated in Table 5. An increase in maize and a decrease in fish had increased the total carbohydrate content (Figure 3F). The ANOVA result indicated the linear model is significant (*p* < 0.05) in describing the changes in carbohydrate due to varying blending ratios (Table 2).

In Table 5, the gross energy value of the flatbread ranged from 334.8 kcal/100 g (MRF‐11) to 356.5 kcal/100 g (MRF‐9) (Figure 3G). The ANOVA result showed that only maize flour had a positive linear effect on the gross energy value of flatbread at *p* < 0.05 (Table 2). Flatbread containing a high gross energy value is desirable to be used as breakfast and complementary foods (Fasasi et al. 2007). Generally, flatbread developed from maize alone contained significantly higher (*p* < 0.05) fat, fiber, carbohydrate, and gross energy value as compared to flatbread developed from composite flours. However, flatbread developed from maize alone contained significantly lower (*p* < 0.05) crude protein and crude ash content as compared to flatbread developed from composite flours.

### 3.4 Mineral Contents

The mineral contents of traditional unleavened flatbread and control (maize alone) are presented in Table 6. The ANOVA result showed that the linear model significantly (*p* < 0.05) described changes in sodium, potassium, iron, and zinc (Table 2). The increase of boiled rhizome of water lily flour and fish flour has tremendously enhanced the mineral content of sodium, potassium, iron, and zinc.

**TABLE 6:** Mineral contents (mg/100 g) of flatbreads.

Sample code	Na	K	Ca	Mg	Fe	Zn
MRF‐1	18.6	320.8	34.2	91.1	2.0	1.9
MRF‐2	43.4	406.1	70.5	102.6	3.0	2.0
MRF‐3	40.9	387.6	61	96.8	2.9	1.9
MRF‐4	24.2	361.4	53.9	102.8	2.3	2.0
MRF‐5	21.4	341.1	44.1	97	2.2	1.9
MRF‐6	59.8	430.5	77.1	96.7	3.7	1.9
MRF‐7	37.8	365.5	50.7	91	2.8	1.9
MRF‐8	40.6	385.8	60.6	96.8	2.9	1.9
MRF‐9	57	410.2	67.2	90.9	3.6	1.9
MRF‐10	40.9	387.4	60.0	96.8	3.0	1.9
MRF‐11	62.6	450.8	87	102.5	3.9	1.9
Maize	0.2	250.3	4	79.6	1.3	1.9

**FIGURE 4:**
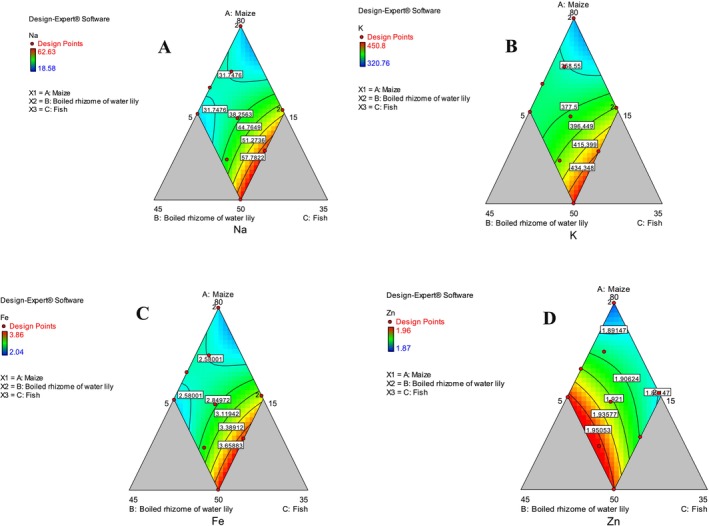
Contour plot showing mineral contents (A—sodium, B—potassium, C—iron, and D—zinc).

Sodium content ranged from 18.58 mg/100 g (MRF‐1) to (62.63 mg/100 g) (Table 6). The results of ANOVA show that the linear model has significantly (*p* < 0.05) described the changes in sodium due to different blending ratios (Table 2). Decreasing the proportion of maize and increasing the ratio of fish have increased sodium content (Figure 4A). Food is claimed to be low in sodium if it contains no more than 0.12 g/100 g, and very low in sodium if it contains 0.04 g/100 g (Regulation (EC) No 1924/2006). Accordingly, all the prepared flatbreads can be considered low sodium and considered very low in sodium except for flatbreads like MRF‐2, MRF‐6, MRF‐9, and MRF‐11.

Potassium was the most abundant element in the prepared flatbreads. The potassium value ranged from 320.76 mg/100 g (MRF‐1) to 450.8 mg/100 g (MRF‐11) (Table 6). The results of ANOVA showed that the linear model had significantly (*p* < 0.05) described the changes in potassium due to varying proportions of ingredients (Table 2). In agreement with the present study, the mineral content of bread developed from the tuber of water lily and wheat was reported to contain potassium (790 mg/100 g) (Ibrahim 2007). Decreasing the proportion of maize and increasing the ratio of fish flour as well as boiled rhizome of water lily flour have increased potassium content (Figure 4B). The high potassium observed in this flatbread is associated with the presence of a high amount of potassium (521 mg/100 g) in the rhizome of water lily (Abelti et al. 2024b).

The recommended daily allowances (RDAs) of potassium are 2000 mg per day, of which 15% of potassium (300 mg/100 g) must be supplied by a specified food if a food is considered to be a source of potassium (Regulation (EC) No 1924/2006). Accordingly, all the prepared flatbreads can supply more than 300 mg/100 g of potassium. The percentage of RDA fulfilled by 100 g of flatbread ranged from 16.0% (MRF‐1) to 22.5% (MRF‐11). Potassium influences many physiological processes in regulating heartbeat, regulating body fluids, lowering blood pressure, preventing kidney stones, maintaining osmotic balance, retaining protein during growth, controlling glucose absorption and insulin metabolism, helping muscle contract, and creating nerve impulses (Samanth 2024).

The iron content ranged from 2.04 mg/100 g (MRF‐1) to 3.86 mg/100 g (MRF‐11) (Table 6). The ANOVA results indicated that the linear models significantly (*p* < 0.05) described the changes in iron due to changing the ratio of three ingredients (Table 2). Increasing the ratio of fish flour and decreasing the ratio of maize have ultimately increased the iron content (Figure 4C).

The body uses iron for a number of essential processes. It functions as a transport medium for electrons within cells, a component of vital enzyme systems (hemoglobin, myoglobin, and cytochromes) in a variety of tissues and a carrier of oxygen from the lungs to the tissues (World Health Organization 2004). The amended Commission Directive 2008/100/EC of the European Union recommends the recommended daily allowances (RDAs) of iron as 14 mg/100 g. The flatbread prepared in the present study can fulfill about 14.5% (MRF‐11) to 27.5% (MRF‐11).

Zinc is a component of several enzymes that support the maintenance of proteins' structural integrity and control the expression of genes (NHMRC 2006). Alcohol dehydrogenase, carbonic anhydrase, alkaline phosphatase, and ribonucleic acid polymerases are typical examples of zinc metalloenzymes. The lowest and highest zinc content was obtained as 1.87 mg/100 g and 1.96 mg/100 g in MRF‐9 and equally in MRF‐2 and MRF‐4 (Table 6). Increasing the ratio of boiled rhizome of water lily and decreasing the ratio of maize have ultimately increased the zinc content (Figure 4D). The ANOVA results indicated that the linear models significantly (*p* < 0.05) described the changes in zinc due to changing the ratio of three ingredients (Table 2). The amended Commission Directive 2008/100/EC of the European Union recommends the recommended daily allowances (RDAs) of zinc as 10 mg/100 g. The flatbread prepared in the present study can fulfill about 18.7% (MRF‐9) to 19.6% (MRF‐2 and MRF‐4).

### 3.5 Overall Sensory Acceptability

**FIGURE 5:**
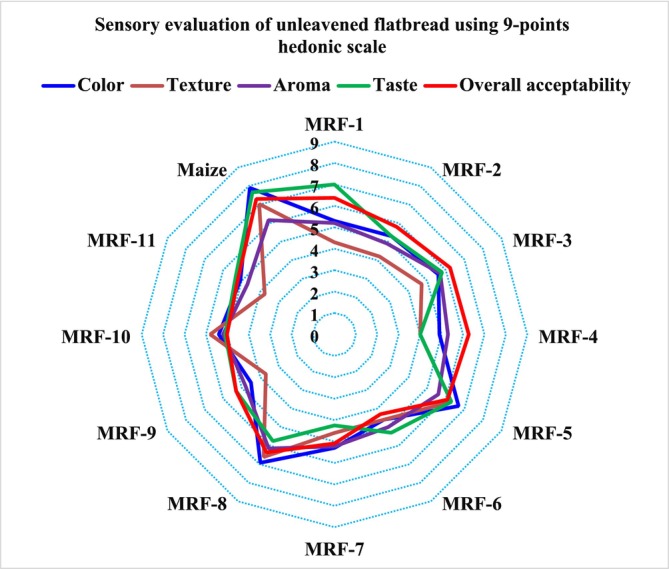
Radar chart of sensory evaluation.

The traditional unleavened flatbreads are prepared from composite flours of maize flour, boiled rhizome of water lily flour, and fish flour. The results from the untrained panelists indicated that there is a difference among the breads in terms of texture, color, taste, aroma, and overall acceptability. The radar chart (Figure 5) revealed the mean scores for the sensory attributes of traditional unleavened flatbread developed from maize flour, boiled rhizome of water lily flour, and fish flour. The most accepted flatbread was the bread developed from maize alone (7.3), followed by MRF‐1 (6.37), MRF‐8 (6.3), MRF‐4(6.27), MRF‐3(6.23), MRF‐5(6.09), and the least accepted bread was MRF‐6 (4.3).

**FIGURE 6:**
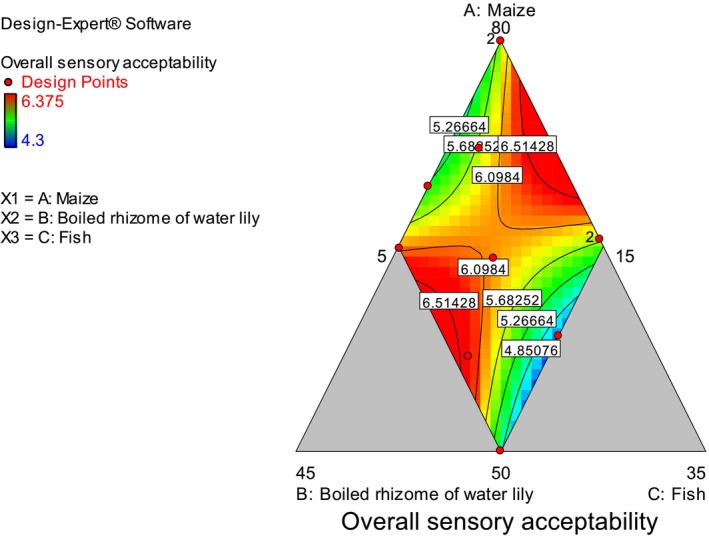
Contour plot of overall sensory acceptability of flatbreads.

The sensory characteristics differed significantly among the flatbreads. The ANOVA result showed that both linear and quadratic models were non‐significant (*p* > 0.05) in describing the changes in overall sensory acceptability due to changing the ratio of maize, boiled rhizome of water lily, and fish flours (Table 2). Even though there is no consistency in the increment or decrement of overall sensory acceptability, an increased proportion of maize, the average ratio of boiled rhizome of water lily, and fish flour had resulted in better overall sensory acceptability (Figure 6). Saeed et al. (2021) observed that the substitution of more than 20% lotus root flour with wheat had resulted in lower scores, harder texture, darker color, and unpleasant flavor. According to Thanushree et al. (2017), a 15% substitution of lotus root powder with wheat flour resulted in a breadstick with an acceptable sensory profile.

In line with the present study, Afrin et al. (2024) observed the incorporation of 20% water lily tuber to wheat for the development of biscuit had the best overall sensory acceptability. In another experiment, the addition of 10% water lily tuber to wheat flour has shown the best overall acceptability of bread (Ibrahim 2007). Recently, chapatti was prepared from fish power and orange‐fleshed sweet potato. It was reported that flatbread prepared from 85 g of Nile tilapia flour and 15 g orange‐fleshed sweet potato had the lowest overall sensory acceptability (7.75) (Mekonnen and Aychiluhm 2024). The optimum selected blending ratio 61.82:38.18 of fish and orange‐fleshed chapatti had been selected in terms of nutritional value (Mekonnen and Aychiluhm 2024).

### 3.6 Numerical Optimization

**FIGURE 7:**
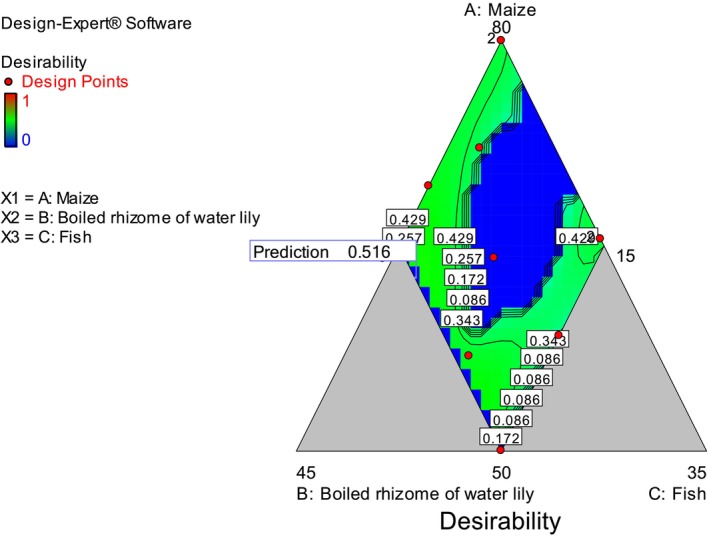
Numerical optimization of functional properties of composite flours, proximate composition, mineral contents, and overall sensory acceptability of flatbreads.

Numerical optimization results showed that a blending ratio containing 62.62% of maize flour, 29.92% of boiled rhizome of water lily flour, and 7.45% of fish flour had resulted in the best formulation with a desirability function value of 0.516 with enhanced protein, gross energy value, potassium, iron, zinc, and overall sensory acceptability of flatbreads (Figure 7). As compared to the present study, a high desirability value (0.9008) was reported for the best nutritional value of chapatti in terms of protein, carbohydrate, beta‐carotene, vitamin A, iron, and magnesium, which was prepared using 61.82% fish and 38.18% orange fleshed‐sweet potato (Mekonnen and Aychiluhm 2024).

### 3.7 Pearson's Correlation

The Pearson's correlation of functional properties, proximate composition, mineral contents, and overall sensory acceptability is presented in Table 7. The water absorption capacity of composite flour had shown significant negative correlation with Carr's compressibility index and gross energy value. Oil absorption capacity had not shown any significant association with all the analyzed parameters. Bulk density had shown a negative correlation with CI and HR. Tapped density had shown a positive correlation with the Hausner ratio. The Carr's compressibility index had a positive correlation with the Hausner ratio and gross energy value. Crude protein content had shown a negative correlation with total fiber, total carbohydrate, and overall sensory acceptability. However, no significant correlation was found between protein content and functional properties of flour. Nevertheless, strong correlations were confirmed to exist between protein and functional properties like WAC and OAC. Some previous studies also reported no correlation between protein and functional properties of gluten‐free flour (Horstmann et al. 2017). Crude fat had positively correlated with gross energy values. A positive correlation had been shown between total fat and energy contents and no correlation between protein, carbohydrate, and gross energy. The previous study reported a strong positive correlation between fat and gross energy of bread products from Italy (Angelino et al. 2020). The total carbohydrate had shown a positive correlation with total fiber and overall sensory acceptability. Carbohydrate is known to improve the textures and structures of baked products, which are desirable characteristics for baked products (Eshak 2016). Gross energy value had shown a negative correlation with water absorption capacity. Iron had shown a negative correlation with the total fiber and total carbohydrate but positively correlated with crude protein and potassium. Overall, sensory acceptability had negatively correlated with crude protein, potassium, and iron but positively correlated with total fat and total carbohydrate.

**TABLE 7:** Pearson's correlation between functional properties of composite flours and proximate composition, mineral content, and overall sensory acceptability of traditional unleavened flatbread.

Code	WAC	OAC	BD	TD	CI	HR	CP	CF	TA	TF	TC	GEV	K	Fe	Zn	OSA
WAC	1.000															
OAC	0.485	1.000														
BD	0.171	−0.008	1.000													
TD	−0.185	0.091	−0.122	1.000												
CI	−0.625*	−0.358	−0.692*	0.275	1.000											
HR	0.285	0.056	−0.692*	0.782**	0.627*	1.000										
CP	0.152	−0.299	0.068	−0.011	−0.102	−0.067	1.000									
CF	0.263	−0.305	−0.187	0.207	0.351	0.351	−0.533	1.000								
TA	0.185	−0.434	0.108	−0.267	−0.273	−0.174	0.270	0.311	1.000							
TF	0.417	0.442	−0.346	−0.015	0.151	0.167	−0.765**	0.243	−0.371	1.000						
TC	0.009	0.414	−0.026	0.106	0.135	0.065	−0.858***	0.147	−0.673*	0.693*	1.000					
GEV	−0.798**	−0.504	−0.241	0.548	0.634*	0.571	−0.070	0.604*	−0.111	−0.142	0.067	1.000				
K	−0.010	−0.211	0.012	−0.115	−0.142	−0.110	0.961***	−0.651*	0.268	−0.698*	−0.808**	−0.273	1.000			
Fe	−0.151	−0.298	0.066	−0.016	−0.105	−0.067	1.000***	−0.531	0.277	−0.765**	−0.861***	−0.073	0.962***	1.000		
Zn	0.469	0.251	−0.213	−0.398	−0.150	−0.159	0.025	−0.460	0.118	0.088	−0.005	−0.733*	0.300	0.031	1.000	
OSA	0.384	0.309	−0.309	0.034	−0.060	0.158	−0.711*	0.233	−0.217	0.657*	0.624*	−0.088	−0.626*	−0.713*	0.177	1.000

*Note:* *Correlation is significant at the level 0.05, **correlation is significant at the level 0.01, and ***correlation is significant at the level 0.001.

Abbreviations: BD, bulk density; CF, crude fat; CI, Compressibility index; CP, crude protein; Fe, iron; GEV, gross energy value; HR, Hausner ratio; K, potassium; OAC, oil absorption capacity; OSA, overall sensory acceptability; TA, total ash; TC, total carbohydrate; TD, tapped density; TF, total fiber; WAC, water absorption capacity; Zn, zinc.

## 4 Conclusions and Recommendations

Traditional unleavened flatbreads developed from maize flour, boiled rhizome of water lily flour, and dried fish fillet powder were rich in protein which has the potential to reduce protein energy malnutrition. The addition of boiled rhizome of water lily flour and dried fish fillet powder increased the crude protein content and total ash content, whereas this addition reduced crude fat, crude fiber, total carbohydrate, and gross energy value as compared to control bread. The crude protein content, total ash, total fiber, total carbohydrate, gross energy value, and major minerals ranged within the permissible values set by the National Institute of medicine, National Health and Medical Research Council of New Zealand and Australia, FAO/WHO, and EU Commission Directive 2008/100/EC for daily dietary reference intake. The presence of high iron and zinc in composite bread as compared to control bread could be advantageous to reduce micronutrient deficiency. The untrained panelists were satisfied with the breads, and the overall sensory acceptability of the prepared flatbread ranged from like slightly to like very much. It can be concluded that boiled rhizome of water lily flour and dried fish fillet powder could be exploited in bread making. It is suggested that additional studies such as shelf stability, sensory analysis in diverse populations, and cost‐effectiveness of this flatbread at large should be carried out before large‐scale utilization.

## Author Contributions

**Alemu Lema Abelti:** conceptualization (equal), data curation (equal), formal analysis (equal), funding acquisition (equal), methodology (equal), writing – original draft (equal), writing – review and editing (equal). **Tilahun A. Teka:** conceptualization (equal), formal analysis (equal), methodology (equal), supervision (equal), writing – original draft (equal), writing – review and editing (equal). **Geremew Bultosa:** conceptualization (equal), formal analysis (equal), methodology (equal), supervision (equal), writing – original draft (equal), writing – review and editing (equal).

## Ethics Statement

This study did not involve any human or animal testing.

## Conflicts of Interest

The authors declare no conflicts of interest.

## Data Availability

The data that support the findings of this study are available on request from the corresponding author.
